# The association between tumour heterogeneity and immune evasion mechanisms in hepatocellular carcinoma and its clinical implications

**DOI:** 10.1038/s41416-024-02684-w

**Published:** 2024-05-17

**Authors:** Kaina Chen, Timothy W. H. Shuen, Pierce K. H. Chow

**Affiliations:** 1 Department of Gastroenterology & Hepatology, Singapore General Hospital, Singapore, Singapore; 2 Duke-NUS Medical School, Singapore, Singapore; 3 Division of Medical Oncology, National Cancer Centre Singapore, Singapore, Singapore; 4 Department of Hepato-pancreato-biliary and Transplant Surgery, National Cancer Centre Singapore and Singapore General Hospital, Singapore, Singapore; 5 Program in Translational and Clinical Liver Cancer Research, National Cancer Centre Singapore, Singapore, Singapore

**Keywords:** Hepatocellular carcinoma, Cancer microenvironment

## Abstract

Hepatocellular carcinoma (HCC) is the third leading cause of cancer-related mortality worldwide. The emergence of combination therapy, atezolizumab (anti-PDL1, immune checkpoint inhibitor) and bevacizumab (anti-VEGF) has revolutionised the management of HCC. Despite this breakthrough, the best overall response rate with first-line systemic therapy is only about 30%, owing to intra-tumoural heterogeneity, complex tumour microenvironment and the lack of predictive biomarkers. Many groups have attempted to classify HCC based on the immune microenvironment and have consistently observed better outcomes in immunologically “hot” HCC. We summarised possible mechanisms of tumour immune evasion based on the latest literature and the rationale for combination/sequential therapy to improve treatment response. Lastly, we proposed future strategies and therapies to overcome HCC immune evasion to further improve treatment outcomes of HCC.

## Background

While hepatocellular carcinoma (HCC) is currently the third most common cause of cancer-related mortalities worldwide [1], the number of new cases and deaths from liver cancer is expected to increase by more than 55% by 2040 [2]. HCC develops almost exclusively from a background of chronic liver inflammation leading to liver fibrosis /cirrhosis that precedes tumourigenesis. Aetiologies of HCC include chronic hepatitis B (HBV) and C (HCV) infection, alcohol-related liver disease, and increasingly, metabolic dysfunction associated-steatotic liver disease. Liver cirrhosis is the final common pathway with an annual risk of 1–8% for developing HCC. One in three cirrhotic patients are expected to develop HCC in their lifetime [3]. Early-stage HCCs are potentially curable with surgical resection, ablative therapies such as radiofrequency or microwave ablation, and liver transplantation. Unfortunately, more than 70% of HCC are diagnosed at a more advanced stage when the overall survival (OS) is less than 30 months [4, 5].

The pathophysiology of HCC is a complex process involving multiple molecular pathways, and the accumulation of molecular alterations over time leads to a heterogenous mutational and epigenetic landscape of the tumour. Unfortunately, known driver mutations in HCC, namely *TERT*, *TP53*, *CTNNB1*, are not targetable by drugs and HCC is well-known for its resistance to systemic therapy [6, 7]. For more than a decade, first-line therapy for advanced HCC has been the multi-kinase inhibitor sorafenib, which only provided <3 months of extended median survival but with notable toxicity [8, 9].

The discovery that immune checkpoint molecules play an essential role in the immune evasion of tumour cells has encouraged clinical trials of immune checkpoint inhibitors (ICIs) in advanced HCC patients. The immune response in cancer is a double-edged sword that can both destroy tumour cells and create an inflammatory microenvironment that enhances tumour progression. Immune checkpoints are mechanisms that control autoimmunity and keep the immune response in check. The U.S. Food and Drug Administration (FDA) granted accelerated approval for pembrolizumab and nivolumab plus ipilimumab as second-line options in patients who progressed from sorafenib based on KEYNOTE-224 and CheckMate 040 respectively [10, 11]. Nivolumab monotherapy failed to demonstrate superior survival benefits over sorafenib in CheckMate 459 [12]. However, only approximately 25% of the HCCs have a robust response to immunotherapy, whereas the majority of HCCs are immunologically “cold” and are associated with an immunosuppressive environment [13]. In 2020, we witnessed a new milestone in the systemic treatment landscape of HCC when a 5.8-month survival advantage over sorafenib was demonstrated with the combination of atezolizumab (anti-PDL1) and bevacizumab (anti-vascular endothelial growth factor [VEGF]) in the IMBRAVE-150 study [14]. Two years later, the combination of durvalumab (anti-PDL1) and tremelimumab (anti-CTLA4) was also approved by the FDA as a first-line treatment of unresectable HCC (without main portal vein invasion) based on the HIMALAYA study [15]. Despite this remarkable success, the best overall response rate with atezolizumab and bevacizumab in advanced HCC was only 30%, and with durvalumab plus tremelimumab was only 20.1% [15, 16]. The failure of ICI monotherapies and the poorly efficacious first-line systemic treatment runs parallel to the absence of validated predictive biomarkers for the selection of a suitable patient population for these therapies, and this poses a critical challenge in HCC clinical management. The complex microenvironment of HCC is pivotal to its poor response to systemic therapy. In this review, we summarise the current evidence on HCC tumour evolution, provide updates on the mechanisms of tumour immune evasion in HCC, and propose rational and strategic combination/sequential therapies to overcome tumour heterogeneity and immune evasion.

## Intra-tumoural heterogeneity (ITH) of HCC

Cancer cells evade the body’s natural defences by acquiring genetic mutations that avoid apoptosis and senescence, promote angiogenesis and metastasis, alter the cellular metabolism to support rapid proliferation with limited nutrients and oxygen supply, and orchestrate a change in the microenvironment to escape immune surveillance [17]. HCC develops from a background of various aetiologies with distinct microenvironments, that stimulated cancer cells to undergo extensive reprogramming at the genetic, epigenetic, and metabolic levels to adapt and sustain growth [18]. The inter- and intra-tumoural heterogeneity of HCC has been described and remained the main interest in the field. With the advent of next-generation sequencing, such tumour heterogeneity has been demonstrated in spatially and temporally separate tumours, with implications in patient prognosis and response to therapeutic agents [19].

Freimel et al. examined 120 tumour areas from 23 treatment-naïve HCC patients and showed that 87% of the cases exhibited intra-tumoural heterogeneity (ITH) in tissue morphology by immunohistochemistry (IHC) and had *TP53* and *CTNNB1* mutations [20]. A more comprehensive study by Zhai et al. used multi-regional sampling of nine resected HCCs from various aetiologies to elucidate the spatial organisation of ITH and constructed a complete clonal evolution map from whole genome/exome sequencing (WGS and WES). All nine tumours followed a clonal branched pattern of evolution, and spatially closer subclones tend to be genetically more similar, exhibiting an isolation-by-distance pattern [21]. A similar study confirmed the clonal evolution pattern of HCC using multi-regional whole-exome sequencing from 11 HCC patients [22]. Zhang et al. incorporated a multi-omic approach (WES, bulk RNA-seq, mass spectrometry-based proteomics and metabolomics, cytometry by time-of-flight [CyTOF] and single-cell analysis) on 42 samples from 8 HCC patients and demonstrated significant heterogeneity in the genomes, transcriptomes, proteomes, and metabolomes of HCC tumours [23]. Using an immunogenomics approach on multi-regional samples of HCC, Losic et al. observed significant regional differences in the magnitude of tumour-infiltrating lymphocytes (TIL) in the tumour microenvironment. Branch mutations, rather than driver mutations, contribute more to the recruitment of TIL, suggesting complex tumour-immune interactions in HCC leading to ITH [24].

The evolution of a tumour ecosystem is driven by selection pressures from the intrinsic instability of the cancer genome as well as the extrinsic environment such as immune regulation and treatment exposure [6]. One important selection pressure in HCC is tumour hypoxia. HCCs are highly vascularised and dysregulated angiogenesis leads to a hypoxic tumour microenvironment [25, 26]. In response to hypoxia, the tumour ecosystem undergoes genetic and metabolic reprogramming [27]. One crucial mechanism of this reprogramming involved the production of hypoxia-induced factors (HIF), acting as a central regulator interacting with Wnt/β-catenin, PI3K/AKT and VEGF pathways, which promote epithelial-mesenchymal transition (EMT) thereby inducing tumour progression and invasion [28, 29]. In addition, hypoxia induces significant changes in the immune microenvironment of HCC, as demonstrated by Suthen et al., who compared hypoxia-high and hypoxia-low regions from the multi-regional sampling of HCC tumour tissues. They found that tumour sectors with high expression of hypoxia-related genes (hypoxia-high regions) had an immunosuppressive tumour microenvironment with the enrichment of exhausted CD8^+^ cells, T regulatory cells (Tregs), and type-2 conventional dendritic cells (DCs), and reduced proportions of active CD8^+^ T cells [30]. Taken together, the above studies demonstrated ITH at the level of immune regulation driven by selection pressures and provided evidence for tumour-immune coevolution in HCC.

## HCC tumour microenvironment

The liver is an immune-privileged organ that maintains the balance between immunotolerance and immune activation by a complex milieu of immune cells and pro- and anti-inflammatory cytokines [31]. The liver receives blood flow from the portal circulation that brings in various bacterial antigens from the gastrointestinal tract, resulting in an enormous antigen exposure and ongoing immune stimulation to the liver [32]. To protect the organ from autoimmune damage, the liver developed immunotolerant mechanisms within both the innate and adaptive immune responses [33, 34].

One of the hallmarks of cancer is the ability to evade the host immune system, allowing continued growth and metastasis [17]. HCC tumour subclones created as a result of ITH are forced to survive by disrupting the immune checkpoint pathways to promote immune evasion [35, 36]. Efforts had been made to classify HCC into different immune subclasses to predict response to immunotherapy. Sia et al. analysed gene expression patterns of inflammatory cells from 956 HCC patients and found that 25% of HCC expressed markers of inflammatory responses, termed the “immune class” [13]. Subsequently, a more comprehensive study using RNA-Seq, WES, T cell receptor sequencing (TCR-seq), multiplex immunofluorescence (mIF) and IHC from 240 patients, followed by validation in other cohorts of 660 patients, further refined immunogenomic classifications of HCC [37]. In brief, three subclasses were proposed. The “*inflamed class*” (37%) consists of the previously reported “immune class” (22%) and an additional 15% of “immune-like” subclass with diverse T cell repertoire and high IFN signalling. The “*intermediate class*” is contributed by tumours enriched in *TP53* mutations and chromosomal losses involving immune-related genes. The last one is the “*excluded class*” mainly enriched in *CTNNB1* mutations and *PTK2* overexpression. Chaisaingmongkol et al. studied liver cancer patients in Asia and identified a molecular subtype (C2), characterised by elevated CD4^+^ memory T cells, reduced Treg cells and higher leucocyte infiltrates, that is associated with good prognosis, suggesting that immunologically “hot” tumours correlated with favourable outcomes [38].

To understand the components of the immune cells in the tumour microenvironment, and their ITH, Kurebayashi et al. analysed 919 tumour sectors from 158 HCCs using multiplexed IHC correlated with histopathological features [39]. They classified the immune microenvironment into immune-high, immune-mid, and immune-low subtypes. The immune-high subtype is characterised by a Th1 cytokine/chemokine milieu and increased PD1/PDL1 expression in CD8^+^ cells. Considerable ITH of the immune cells was observed, but the predominant immune subtype was prognostically important. Subsequently, Nguyen et al. used multiple tumour sectors to demonstrate that immune heterogeneity was closely associated with mutation burden, transcriptomic-ITH, immunosuppressive/exhausted tumour microenvironment. Tumours with high immune-ITH positively correlated with immunosuppressive and immune exhausted cells, but negatively correlated with cytotoxic and activated immune cells, suggesting that high immune-ITH was linked to an exhausted TME. Clinically, high immune-ITH correlated with poor prognosis [40]. Taken together, the findings indicate that tumour-infiltrating immune cells play a crucial role in inhibiting tumour progression. Tumours with increased immune infiltrates (“hot” immune microenvironment) are associated with more favourable outcome, and evasion of immune mechanisms leads to poor survival outcomes and compromised susceptibility to immunotherapy.

However, it is important to recognise that even in highly immune infiltrated HCCs, immune evasion mechanisms such as modulation of the abundance of immunosuppressive immune cells and reduced expression of major histocompatibility complex (MHC) may still be at play [40, 41]. Other mechanisms such as oncofoetal reprogramming, and epigenetic modulations of the immune cells are emerging as new mechanisms of tumour microenvironment interactions. We will discuss these mechanisms in the following sections of this review.

## Oncofoetal reprogramming

Tumour cells are known to exhibit phenotypic plasticity that facilitates tumour cell evolution [17]. This has often been compared to embryonic development in the context of the re-expression of foetal proteins in malignant tumours, termed oncofoetal antigens [42]. Oncofoetal antigens have been identified in several tumour types and are now utilised clinically as biomarkers for cancer surveillance, diagnosis, and prognosis [43, 44]. One of the best-characterised tumour markers in HCC is alpha-fetoprotein (AFP). AFP is a human glycoprotein whose main physiological function is the regulation of the entry of fatty acids into foetal and proliferating adult cells via the AFP receptor-mediated autocrine system [45]. AFP is frequently used in combination with imaging for surveillance with a view to early diagnosis of HCC in patients with chronic liver disease and for recurrence of HCC treated with ablative therapy. In addition, AFP is used for prognostication and selection for liver transplant candidates, as high levels of AFP are correlated with poor differentiation of the tumour and generally poorer clinical outcomes [45, 46]. Another oncofoetal antigen for HCC is SALL4, an active nuclear factor during embryonic development which is instrumental in maintaining stem cell pluripotency [47]. Although there are fundamental differences between tumourigenesis and embryogenesis, they share a remarkable resemblance in their tolerance to the immune microenvironment [48, 49]. To test the hypothesis that the foetal-like tumour microenvironment displays immunosuppressive properties that helped the tumour evade our immune surveillance, Sharma et al. profiled foetal-liver, HCC tissues, and adjacent non-tumour liver tissues using 96 immune, stromal, epithelial and oncofoetal markers, and incorporated multi-regional sampling of the tumour tissues with a spatial transcriptomics approach. They found that foetal-liver and HCC both exhibit classic immunosuppressive T cells (FOXP3, CTLA4, LAG3, BATF3) and the re-emerged foetal-associated endothelial cells and foetal-like TAMs in HCC co-cluster with one another. Furthermore, spatial transcriptomic data highlighted a co-localisation of VEGF-NOTCH signalling and the interactions among the foetal-associated *PLVAP*^+^ /*VEGFR2*^+^ endothelial cells, embryonic-like *FOLR2*^+^ /*CD163*^+^ TAMs and immunosuppressive regulatory T cells (CTLA4) that maintained an immunosuppressive tumour microenvironment in HCC [48]. This discovery offers profound insights to clinical management as it provided a potential explanation of the mechanisms of atezolizumab (anti-PDL1) and bevacizumab (anti-VEGF) in HCC treatment and suggested the possibility of utilising oncofoetal biomarkers to individualise treatment selection.

## Epigenetic modulation of the immune cells

The epigenome is an inheritable trait that determines transcriptional output without changes in the DNA sequences or genetic mutations [50]. Epigenetic silencing of immune-related genes has been recognised as an important mechanism contributing to carcinogenesis [51]. Studies focusing on epigenetic regulations of the immune checkpoints revealed an important interplay between immune modulation and different epigenetic mechanisms including DNA methylation, histone modification, micro-RNAs (miRNAs) and long non-coding RNAs (lncRNAs) [51]. DNA methylation is one of the most studied epigenetic phenomena in cancer research. In most cancers, hypermethylation of the cytosine residues to 5-methylcytosine by DNA methyltransferases (DNMTs) at the cytosine-guanine dinucleotides (CpG) islands within the promoters, leads to the silencing of various tumour suppressor genes [52–54]. While hypermethylation of the CTLA4 promoter has been associated with increased gastric cancer risk, the aberrant methylation pattern at the locus encoding PD1, PDL1, and PDL2 can be reversed with the use of the demethylating agent decitabine to enhance immune checkpoints expression and has been used in the treatment of myelodysplastic syndrome [55, 56]. Liu et al. observed that rather than mutations and copy number variations (CNVs), epigenetic modifications such as global methylation patterns and miRNA sponges seem to play a crucial role in HCC immunomodulation [57]. One of the immune subtypes of HCC described by Montironi et al., the “*immune excluded*” class, was characterised by PTK2 overexpression, which was associated with promoter hypomethylation, suggesting epigenetic modifications as one of the tumour immune escape mechanisms [37]. Using a bioinformatic approach on the TCGA database, Xu et al. classified HCC patients based on a prognostic signature generated from the DNA methylation level at the CpG islands, which had a strong correlation with tumour immune microenvironment and ICI-related genes [58]. There are limited studies on histone modifications in HCC tissues and its derangement related to immune modulations in HCC. Earlier studies using immunohistochemistry suggested specific histone modifications associated with HCC prognosis [59, 60]. More recently, Jeon et al. studied aberrant epigenetic events in HCC, focusing on the acetylation of H3K27 (H3K27ac) which marks gene enhancers. Large-scale changes in the enhancer distribution between HCC and non-tumour liver tissues were observed, and the patient cluster with a poor prognosis based on enhancer signature also had downregulated expression of the immune defence response [61].

## Impact of immune evasion on treatment outcomes

The FDA approval of immunotherapy-based treatment for advanced HCC has spurred investigations for biomarkers that can predict treatment outcomes for ICI. Harding et al. analysed genomic alterations in 127 HCC samples and attempted to correlate with various systemic treatments to identify the genomic biomarkers of response and resistance. They found that only HCCs with altered Wnt/β-catenin including mutations of *CTNNB1* and *AXIN1* correlated with worse clinical outcomes in 31 patients who received ICI mono- or combination therapy, and no other pathways correlated with ICI responsiveness [62]. Separately, Galarreta et al. developed novel genetically engineered HCC mouse models to study how genetic alterations affect immune surveillance and response to immunotherapy [63]. The study revealed that immunotherapy is effective in suppressing tumour formation in *Myc*;*Trp53*^-/-^ HCC model, and such tumours escaped the immune system by activating β-catenin pathways. Further testing of immunotherapy in a *Myc*;*Ctnnb1* HCC model showed that defective DCs are recruited to the tumour to inhibit T-cell activity, which could be rescued by expression of *Ccl5*, leading to better tumour control. Collectively, these two studies suggested that altered β-catenin signalling activation may confer resistance to anti-PD1 monotherapy in HCC. Wong et al. showed that 33% of non-alcoholic fatty liver disease (NAFLD)-HCC harbour β-catenin mutation and their preclinical testing supported the blocking of mutated β-catenin-mediated immune exclusion for better tumour regression with ICI [64]. Nonetheless, a landmark molecular biomarker study leveraging on the tissue samples collected from the GO30140 phase 1b and the IMbrave150 showed that *CTNNB1* mutation was not a significant prognostic factor in patients who received combination atezolizumab and bevacizumab. It suggests that the addition of an anti-VEGF agent (bevacizumab) may eradicate tumour cells bypassing the ICI resistance from β-catenin activated HCCs [65]. Due to the discrepancy between preclinical and clinical studies, it remains controversial regarding the association between β-catenin mutation and ICI treatment outcomes.

Pfister et al. performed a meta-analysis of three landmark randomised controlled phase 3 trials (IMBrave150, CheckMate 459, and KEYNOTE-240) [66]. It showed that patients with non-viral HCC derived lesser benefit from anti-PD1 or anti-PDL1-based immunotherapy than patients with HBV/HCV-HCC. The team further provided proof-of-concept preclinical data to suggest the impaired immune surveillance in non-alcoholic steatohepatitis (NASH)-HCC and shed light on the aetiology-related immune evasion mechanisms on treatment outcomes [66]. The distinct response rate from IO-based therapy was however not demonstrated in the latest adjuvant trials, namely IMbrave050 where atezolizumab-bevacizumab post-resection/ablation was tested, and EMERALD-1 trial where durvalumab ± bevacizumab post-TACE was evaluated. Both studies did not observe any different response rates between viral and non-viral aetiology of HCC [67, 68]. In summary, there is insufficient evidence to support aetiology-specific HCC management strategies for viral and non-viral HCC. Further translational research will be necessary to dissect the specific impact of HCC aetiologies on the tumour microenvironment and how these differences influence the response to various HCC therapies.

The translational study by Zhu et al. discovered that the combination of atezolizumab and bevacizumab achieved better clinical responses from HCC tumours with pre-existing anti-tumoural immunity, lower Treg to effector T cell (Teff) ratio, and lower expression of oncofoetal genes (GPC3, AFP) [65]. In addition, it suggested that the combination treatment is better than atezolizumab alone in HCCs with high expression of VEGF receptors, Treg and myeloid inflammation signatures. Single-cell transcriptomic analysis of tumours collected at the baseline before immunotherapy or on-treatment from 19 patients revealed that the tumours with high ITH are associated with higher expression of VEGF and cancer stem cell-related genes, which in turn promotes TME reprogramming and affects T cell cytolytic activities [69]. More recently, Haber et al. studied the molecular and immune markers that predict response to ICI in advanced HCC. They generated an 11-gene signature including enhanced interferon-γ signalling and MHC II-related antigen presentation that predict response and survival in patients treated with anti-PD1 as first-line therapy but could not predict response in patients who received first-line tyrosine kinase inhibitors (TKIs) prior to ICI [70].

**Fig. 1:**
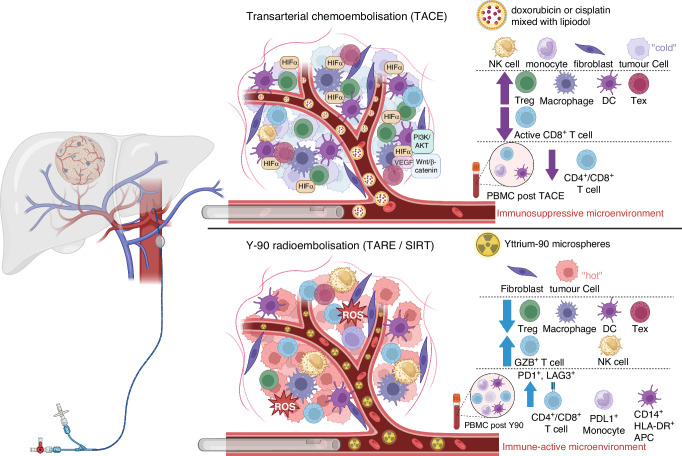
Tumour immune microenvironment alterations post transarterial chemoembolisation versus Y-90 radioembolisation. TACE transarterial chemoembolisation, TARE transarterial radioembolisation, Y-90 yttrium-90, SIRT selective internal radiation therapy, PBMC peripheral blood mononuclear cells, HIF hypoxia-induced factors, Treg regulatory T cells, DCs dendritic cells, Tex exhausted T cells, NK cell natural killer cell, GZB granzyme B, APCs antigen-presenting cells, ROS reactive oxygen species.

Loco-regional therapy is the standard of care for intermediate-stage HCC and serves to reduce and control disease burden and prolong survival. Transarterial treatment modalities, such as transarterial embolisation (TAE), chemoembolisation (TACE), and radioembolisation (TARE), are widely used for this group of patients. Despite similar treatment delivery modalities, the tumour biology post-treatments are distinctly different with these therapies. TACE involves the administration of chemotherapeutic agents (doxorubicin or cisplatin) mixed with lipiodol to obstruct the tumour-feeding arteries, which leads to tumour hypoxia and ischaemic necrosis [71, 72]. Considering the findings of Suthen et al. [30], it is reasonable to hypothesise that TACE-induced tumour hypoxia will lead to an upregulation of hypoxia-related genes, which in turn will create an immunosuppressive tumour microenvironment potentially rendering post-TACE ICI monotherapy less effective. This hypothesis is consistent with the results of the following studies. Matsui et al. studied the histology of 6 resected HCC specimens 2-8 weeks post TACE treatment and found no to minimal inflammatory infiltrates in the resected liver specimens, suggesting the lack of significant immune response with tumour ischaemic necrosis [73]. Pinato and colleagues examined the impact of TACE on HCC tumour immune infiltrates in 119 patients who underwent liver resection/transplantation with or without prior TACE. Their findings again revealed a significant decrease in intra-tumoural T cells, specifically CD4^+^/FOXP3^+^, CD8^+^ and CD8^+^/PD1^+^ T cells, in patients who received TACE before surgery compared to those who did not receive TACE [74]. A study evaluating the peripheral blood mononuclear cells (PBMC) of post-TACE patients also revealed decreased level of CD4^+^/CD8^+^ cells [75] (Fig. 1). A recently published landmark clinical trial investigating the efficacy of adjuvant atezolizumab and bevacizumab post-liver resection or radiofrequency ablation (RFA) (IMBrave050 trial) interestingly showed that patients who received TACE post-surgical resection/RFA before subsequently receiving adjuvant atezolizumab and bevacizumab did worse than those who did not receive TACE [67]. Tumour hypoxia is one specific aspect of tumour biology modification influenced by TACE but we do not fully understand yet the temporal TME changes after TACE and when opportunities may arise to improve tumour hypoxia post-TACE. Several phase 2/3 trials are currently ongoing to evaluate the efficacy of combination TACE and immunotherapy in intermediate-advanced HCCs, and their results are highly anticipated. The recent readout of the EMERALD-1 trial, a double-blinded phase 3 study evaluating the benefit of additional durvalumab (anti-PDL1) / bevacizumab to TACE, was presented in the American Society of Clinical Oncology (ASCO) GI conference 2024 and described improved progression-free survival (PFS) in patients who received combination durvalumab and bevacizumab, but not durvalumab alone, after TACE although durvalumab monotherapy post-TACE showed an improved objective response rate compared to TACE alone [68]. This suggests the addition of an anti-VEGF agent (bevacizumab) normalised abnormal angiogenesis, increased tumour infiltration of the immune cells and acted as an immunostimulatory agent in combination with ICI to overcome the hypoxic TME post-TACE. However, nearly half (46.9%) of the trial patients had relatively small tumour burden (within up-to 7 criteria) and treatment-related mortality rates in the treatment arms are significantly high (9.1% in D + TACE, 10.4% in D + B + TACE, versus 5.5% in TACE alone). We look forward to the OS data and results from the translational arm of this study to shed light on the potential predictive biomarkers of this combination therapy.

Another therapy, Yttrium-90 (Y-90) radioembolisation (also termed selective internal radiation therapy, or SIRT) is used as a loco-regional treatment for intermediate-advanced HCC including HCC with portal vein invasion, which has repeatedly demonstrated very good efficacy and safety profile [76, 77]. As Y-90 microspheres are much smaller than particles used in TACE, there is minimal arteriole embolisation following therapy hence the tumouricidal effects are primarily mediated by radiation injury rather than ischaemic changes. Two independent groups evaluated tumour samples after Y-90 radioembolisation and confirmed that the treatment led to an increased immune activation [78, 79]. Chew et al. studied 41 HCC patients treated with Y-90 radioembolisation including patients who had subsequent liver resection after downstaging. The immune landscape of tumour samples was analysed by CyTOF and NGS, and Y-90 radioembolisation treated tumours were found to have higher granzyme B (GZB)^+^, CD8^+^ T cells, CD56^+^ NK cells and CD8^+^ CD56^+^ NKT cells. Upregulation of genes of innate and adaptive immune activation was also found in Y-90 treated tumours compared to controls, suggesting a local immune activation. Comparing pre- and post-radioembolisation PBMC samples, an increase in tumour necrosis factor-α (TNFα)^+^ CD8^+^ and CD4^+^ T cells were found post Y-90 treatment, suggesting an increased systemic immune activation [78, 79]. Rivoltini et al. subsequently confirmed from peripheral blood samples the increased frequency of activated CD3^+^ T cells and CD8^+^ subsets, Treg, and inflammatory monocyte populations (PDL1^+^, HLA-DR^+^) in Y-90 treated patients [79]. The frequency of PD1^+^ CD3^+^ T cells peaked at 1 month after Y-90 radioembolisation and rapidly returned to baseline within 3 months. Patients with better clinical outcomes had higher levels of GZB^+^ Ki67^+^ CD4^+^ T cells induced after Y-90 treatment (Fig. 1). In addition, Craciun et al. compared intra-tumour immune infiltrates in resected HCC after preoperative treatment with TACE or Y-90 radioembolisation. Consistent with above studies, there is a significant increase in TILs and GZB expression in resected HCC after Y-90 radioembolisation, especially in patients receiving >100 Gy [80]. No differences in immune infiltrates were observed in samples from patients with or without TACE prior to surgery. Collectively, their work suggested that Y-90 radioembolisation alters the tumour biology and creates an immune “hot” tumour microenvironment that potentially augments the response of subsequent immunotherapy. If sequential therapy is considered, the window of opportunity for administering immunotherapy should be between 1-3 months post Y-90 radioembolisation. Tai and colleagues conducted a single arm, single centre, phase 2 clinical trial of Y-90 radioembolisation followed by anti-PD1 treatment (nivolumab) in 36 patients with advanced HCC including those with extrahepatic metastasis [81]. Nivolumab was administrated to patients 21 days after Y-90 radioembolisation and continued every 2 weeks thereafter. Although an encouraging objective response rate of 30.6% was observed (95% CI 16.4–48.1), it was not as high as the study was powered for. A similar single-arm phase 2 study, NASIR-HCC, evaluated the same combination in patients with intermediate-advanced HCC without extrahepatic metastasis, and reported an overall response rate of 41.5% (95% CI 26.3–57.9) with an acceptable safety profile [82]. There are currently two randomised phase 2 trials evaluating the safety and efficacy of Y-90 radioembolisation followed by atezolizumab and bevacizumab: one in locally advanced HCC with comprehensive translational analysis as exploratory objectives (NCT05377034), and another open-label trial evaluating Y-90 followed by durvalumab with tremelimumab (NCT05063565). The outcomes of these trials will inform the clinical response of this sequential therapy and shed light on putative predictive biomarkers. Overall, these research findings enhance our understanding of the intricate interplay between the immune system and the tumour microenvironment in HCC, providing valuable insights into optimising therapeutic strategies for better treatment outcomes.

Currently, two IO-based systemic therapies were approved as first-line for advanced HCC: atezolizumab-bevacizumab and durvalumab-tremelimumab. Based on the objective survival and response data, the first choice would still be atezolizumb-bevacizumab because of the robust hazard ratio on the OS (0.66) and PFS (0.65) compared to sorafenib, and a median OS of 19 months [16]. Durvalumab-tremelimumab demonstrated a median OS of 16.4 months with a hazard ratio of 0.78 on the OS compared to sorafenib in advanced HCC patients without main portal vein thrombosis but without any PFS benefits [15]. At the moment, it can be considered as an alternative in patients with contraindications to bevacizumab. Further studies will be required to look for predictive biomarkers to select patients for each permutation of the HCC  therapies.

## Future directions

### Evolving technologies are driving clinical and translational research

The strategic integration of evolving technologies with well-defined hypotheses and the applications of these technologies in clinical settings, will yield fresh perspectives and lead to transformative breakthroughs and novel discoveries. One major challenge in the field is insufficient tumour samples prior to HCC treatment, and the intrinsic limitation of percutaneous tumour biopsy samples in light of the highly heterogeneous nature of HCC. Firstly, non-invasive diagnosis of HCC can be established in at-risk patients based on the characteristic “arterial enhancement and delayed washout” imaging feature without histological confirmation, with a sensitivity of 89% and specificity of 95% [83, 84]. In this, HCC is quite unlike other common cancers such as breast, lung or colorectal cancers. Secondly, obtaining biopsy samples from treated patients may be clinically challenging, as the procedure confers risks and is currently of no therapeutic value and a single biopsy sample may not be representative of the underlying tumour biology given the high intra-tumoural heterogeneity (ITH) as discussed previously. Most of the ITH studies relied on multi-regional sampling of resected specimens from patients with non-metastatic HCC [20, 21, 24]. However, recent advancements in single-cell technology have suggested that analysing biopsy samples may be a feasible approach and may correlate with clinical responses to immunotherapy in HCC [85]. Advanced technologies and bioinformatic analytic pipelines are facilitating the integration of multi-omics data, including single-cell and spatial copy number variation (CNV), genomics, transcriptomics, TCR clonality analysis, metabolomics, secretomics, and multiplexed immunofluorescence-based image analyses. For example, combined spatial transcriptomics with single-cell RNA-Seq and mIF identified previously unknown phenotypes of tumour immune barrier in the HCC tumour microenvironment that correlate with immunotherapy efficacy [86]. Another integrated analysis of histopathological examination, mutational analysis, single-cell RNA-Seq, single-cell TCR-Seq, and spatial TCR imaging revealed the unique immunophenotypes of CXCL13^+^ PD1^+^ CD4^+^ T helper and GZMK^+^ PD1^+^ effector-like CD8^+^ cells in the HCC tumour microenvironment of responders treated with neoadjuvant anti-PD1 [87]. In contrast, non-responders showed higher levels of terminally exhausted CD39^hi^ TOX^hi^ PD1^hi^ CD8^+^ T cells in the tumours. The subsequent single-cell and spatial analysis led to the discovery of cellular triads of CXCL13^+^ CD4^+^ T cells, DCs with maturation and regulatory molecules, and tumour-specific progenitor exhausted CD8^+^ T cells in the tumour of responders. In summary, these technological advancements are pushing the boundaries in studies on tumour and immune heterogeneity where there are limited tissue samples. Prospective translational and biomarker studies in a clinical trial setting are crucial to understanding the underlying mechanisms of response and resistance. A phase 2 multi-institutional clinical translational study leveraging on surgically resected HCCs of patients subsequently treated with adjuvant atezolizumab plus bevacizumab has been recently initiated, with the aim of elucidating the complex interplay between HCC microenvironment and response to immunotherapy (NCT05516628).

### Clinical molecular imaging modalities in HCC research

There has been insufficient use of clinical imaging modalities to study and track molecular biomarkers in real time in patients undergoing systemic and loco-regional therapies in HCC. Specific T-cell populations can be identified and tracked in patients in real time but this has not been used in clinical studies in HCC [88, 89]. T-cell imaging with 2′-deoxy-2′-18F-fluoro-9-β-d-arabinofuranosylguanine (18F-AraG) is now being evaluated clinically in hematopoietic stem cell transplant recipients (NCT03367962). Similarly, although the technology is already available, the application of radiogenomics in HCC studies is poorly developed [90]. An et al. used RNA sequencing and whole-exome sequencing data obtained from 117 patients with HCC who underwent hepatic resection with preoperative FDG-PET/CT imaging as a discovery cohort for radiogenomic signatures, which were then validated with transcriptomes from a second cohort of 81 patients with more advanced tumours. They found upregulation of mTOR pathway signals in FDG-avid tumours and that treatment with an mTOR inhibitor resulted in decreased FDG uptake followed by effective tumour control in both hyperglycolytic HCC cell lines and xenograft mouse models [91]. This suggested a functional imaging-guided treatment for HCC. Although a randomised clinical trial using everolimus as the second-line treatment of advanced HCC did not demonstrate a survival advantage (EVOLVE-1), subsequent studies have highlighted the benefits of mTOR inhibitor in reducing HCC recurrence in post-transplant patients [92–94].

Used in tandem with translational multi-omics studies, such real time clinical imaging of biomarkers in patients before and during therapy may well provide the breakthroughs needed in our search for validated predictive biomarkers in a cancer with such high intra-tumoural heterogeneity as HCC.

## Strategic approaches to overcome immune evasion mechanisms in HCC

**Fig. 2:**
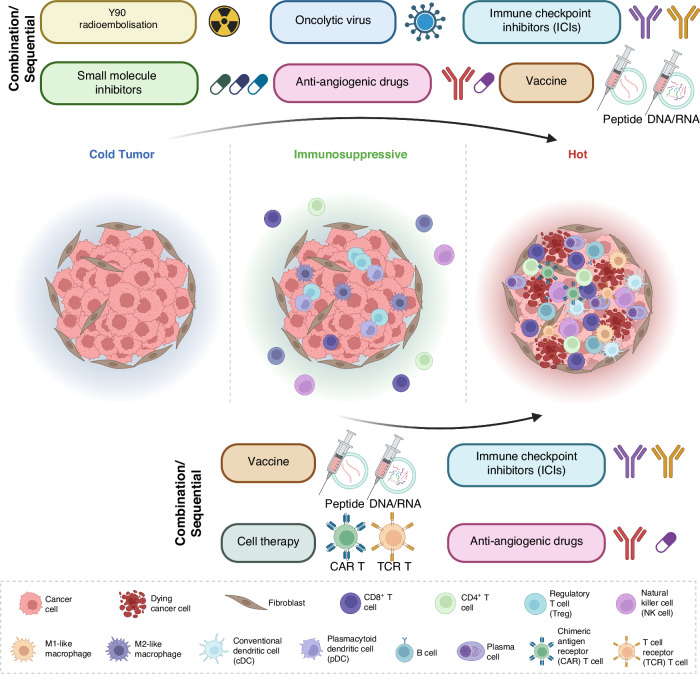
Strategic approaches for overcoming immune evasion mechanisms in Hepatocellular Carcinoma (HCC) involve transforming the immune cold tumour microenvironment (TME) into an immune hot TME. Additionally, efforts focus on converting immunosuppressive, pro-tumour TME into an effective, anti-tumour immune hot TME. Treg regulatory T cell, NK cell natural killer cell, cDC conventional dendritic cell, pDC plasmacytoid dendritic cell, CAR T chimeric antigen receptor T cell, TCR T T cell receptor T cell.

The constant adaptation of the tumour microenvironment in response to therapy is a specific challenge in HCC in view of the significant intra-tumoural heterogeneity which potentially induces the expansion of non-responding clones. A few strategic approaches are suggested below (Fig. 2).

- Calibrating and modulating the immune microenvironment: this includes the utilisation of small molecule protein kinase inhibitors currently under preclinical testing, such as FGFR inhibitor, TGFβ inhibitor, etc [95]. The primary goal is to target activated CTNNB1 pathways or *CTNNB1* mutations to induce immune infiltration and/or to target highly expressed VEGF to induce vessel normalisation, thus improving tumour hypoxia and facilitating higher immune infiltration. However, further pre-clinical and clinical studies are required to evaluate the effectiveness and safety of such strategy in advanced HCC. Additionally, given the historical poor performance of TKI-based combinations in targeting HCC over the past decades, it is crucial to have a rational design for the application and development of new small molecule inhibitors guided by ITH and immune evasion studies.
- Cell Therapy: adoptive T cell transfer involves isolating patients’ immune cells, modifying and/or expanding them ex vivo, and infusing the engineered T cells back to the patients. The most commonly tested approach is chimeric antigen receptor (CAR) T cells. CAR-T has been widely tested in liquid tumours including leukaemia, lymphoma, and myeloma and achieved favourable clinical outcomes. Considerable efforts are being made to target the overexpressed AFP, CD133, GPC3, and MUC1 antigens on HCC tumour cell surface (NCT05003895 and NCT02587689) [96–98]. CAR-T therapy is particularly useful for HCC tumours that have an absence or lower expression of MHC class I and II due to the loss of heterozygosity of human leucocyte antigens (HLAs) or suppression of their RNA expression.
  Alternatively, the group led by Bertoletti et al. proposed using integrated HBV-DNA expression profiles to select TCRs to engineer autologous T cells in the form of TCR-T cells [99]. A clinical study using these TCR-T cells showed long-term clinical benefit, suggesting a new form of cell therapy for HCC patients [100]. Another group developed improved TCR-T against HLA-A2 restricted AFP epitope (AFP158), leading to better tumour infiltration and persistence in the tested preclinical mouse models [101]. However, the main disadvantage of using TCR-T is the HLA-dependent cell killing and current developments are mostly HLA-A2 restricted.
- Cancer vaccine: the paucity of tumour-reactive T cells is one important immune evasion mechanism in cancer. Vaccination plays a crucial role in active immunity, aiding in the generation of a higher frequency of tumour-reactive T cells [102, 103]. Various cancer vaccine approaches have been tested in HCC. For instance, tumour lysate-pulsed autologous DCs were tested in advanced HCC patients and showed no significant toxicity [104, 105]. Butterfield et al. tested four AFP peptides pulsed autologous DCs in 10 HCC patients and successfully increased the number of IFNγ-producing AFP-specific T cell responses [106]. More recently, Cai et al. tested neoantigen long-peptide vaccines in 10 HCC patients with portal vein tumour invasion who underwent radical surgery followed by prophylactic TACE, and subsequently received personalized neoantigen vaccines [107]. No adverse events were observed, and 5/7 patients who received all planned vaccines demonstrated neoantigen-induced T-cell response and longer recurrence-free survival. The collective evidence has reignited interest in cancer vaccines for precision medicine in HCC. Our centre has recently initiated a clinical translational study to evaluate the efficacy and safety of prophylactic neoantigen-pulsed DC vaccine plus nivolumab after HCC resection to prevent postoperative recurrence (NCT04912765).
- Combination/Sequential therapy: single-agent ICIs have limited efficacy in treating advanced HCC compared to sorafenib alone. We have described the changes in the tumour immune microenvironment after Y-90 radioembolisation and the rationale for combining Y-90 with immunotherapy in the ongoing clinical trials (NCT05377034 and NCT05063565). Next-generation therapies should focus on combination/sequential treatment, such as loco-regional followed by systemic therapy, combination systemic therapy including double or triple anti-PD1/PDL1-based treatment, ICI combined with newer treatments such as protein kinase inhibitors, epigenetic modulators, cell therapy or cancer vaccines. The goal is to mitigate tumour cell immune evasions within the heterogeneous nature of HCC while preserving liver function as much as possible. A promising trial testing triplet combination in unresectable, locally advanced or metastatic HCC (the MORPHEUS-liver study, NCT04524871) showed that tiragolumab (anti-TIGIT antibody) in combination with atezolizumab plus bevacizumab achieved higher objective response rate and longer progression-free survival with comparable safety profiles while pending overall survival data [108]. Another proposed IO combination of CXCR2 (receptor crucial to neutrophil recruitment and highly expressed in NASH-HCC) and anti-PD1 treatment aimed at inhibiting tumour-associated neutrophils demonstrated reprogramming of the tumour immune microenvironment that promotes ICI in NASH-HCC [109].

## Conclusion

In the rapidly evolving landscape of HCC therapies, combination immune-oncology therapies (atezolizumab plus bevacizumab, durvalumab plus tremelimumab) demonstrated remarkably improved survival over sorafenib in patients with advanced HCC. Disappointingly, despite breakthroughs in HCC treatments, the best overall response rate was only 30% [16], likely contributed by extensive tumour heterogeneity [35, 110]. Trials on combinations of systemic therapy, and sequential loco-regional and systemic therapies are ongoing in an attempt to improve clinical outcomes in HCC. HCC cell evolution and immune evasion are complex processes that significantly impact on the prognosis of the disease. Currently, the major challenge to better clinical outcomes in HCC remains the absence of validated predictive biomarkers to allow personalised treatment regimens for optimal treatment response. This is hampered by the limited ability of percutaneously obtained biopsy samples to undercover biomarkers. However, with advances in technology and a better understanding of the biology of HCC, there is every hope that new treatments and strategies will emerge to significantly improve clinical outcomes for patients with HCC.
